# Synthesis of High-Performance CSA Cements as Low Carbon OPC Alternative

**DOI:** 10.3390/ma14227057

**Published:** 2021-11-20

**Authors:** Bogdan-Catalin Marin, Georgeta Voicu, Stefania Stoleriu

**Affiliations:** Department of Science and Engineering of Oxide Materials and Nanomaterials, Faculty of Applied Chemistry and Materials Science, University Politehnica of Bucharest, 1-7 Gheorghe Polizu Street, 011061 Bucharest, Romania; marin.bogdan.catalin@gmail.com (B.-C.M.); georgeta.voicu@upb.ro (G.V.)

**Keywords:** calcium sulphoaluminate (CSA) cements, hydration, raw materials, compressive strength

## Abstract

Starting from natural raw materials, cements based calcium sulphoaluminate (CSA) clinkers have been successfully obtained as an eco-friendly alternative to ordinary Portland cement. CSA-based cements with ye’elimite as the main phase have been produced over the years and are widely used today. In this regard, the present paper considers the study of hydration processes for CSA pastes prepared with a water/cement ratio of 0.5 according to the EN-197 standard and their characterization by thermal analysis (DTA-TG), X-ray diffraction analysis (XRD), and scanning electron microscopy coupled with energy dispersive X-ray spectroscopy (SEM-EDX). A mechanical strength of 60.9 MPa was the greatest achieved for mortars hardened for 28 days.

## 1. Introduction

Calcium sulphoaluminate (CSA) cements are a potential new class of low-CO_2_ emission binders compared to ordinary Portland cements (OPC) for environmentally friendly building materials [1,2,3], possessing high early-strength properties, fast setting time, and high amounts of amorphous phases [4,5]. On the one hand, the hydration process of gypsum at early stages leads to ettringite and aluminum hydroxide formation [6,7]. On the other hand, supplementary addition of calcium hydroxide favors the ye’elimite hydration reaction, thus producing a sulfate/hydroxy solid solution AFm phase at early ages and ettringite at later stages [8,9]. The mentioned reactions are described by the following equations:

(1) $$C_{4}A_{3}\$\;+18H\;\to {\;C}_{3}A\cdot C\$\cdot H_{12}+2{\mathrm{AH}}_{3}$$

(2) $$C_{4}A_{3}\$+2C\$+34H\;\to {\;C}_{3}A\cdot 3C\$\cdot H_{32}+2{\mathrm{AH}}_{3}$$

(3) $$C_{4}A_{3}\$\;+8C\$+6\mathrm{CH}+34H\;\to \;3\left(C_{3}A\cdot 3C\$\cdot H_{32}\right)$$

where: C—CaO; A—Al_2_O_3_; $—SO_3_; H—H_2_O.

The calcium sulphoaluminate clinker to calcium sulphate ratio determines the ettringite to monosulphoaluminate ratio in the final product, which increases with increasing calcium sulphate addition. The maximum reachable ettringite content corresponds to an addition of calcium sulphate of about 30% [10,11]. 

This paper’s aim is to investigate further possibilities of obtaining calcium sulphoaluminate clinker, which can be produced starting from natural raw materials such as limestone, clay, gypsum, and bauxite. The clinker composition and gypsum addition have a significant impact on cement mechanical properties and setting time compared to Portland cement.

On the other hand, in contrast to the OPC, which causes the release of 5–7% of the total CO_2_ emissions into the atmosphere [12,13], the CSA cements containing ye’elimite, belite and anhydrite or ferrite, respectively, can lower the CO_2_ level released in the environment by 30% [14,15]. Furthermore, considering the reduced amount of limestone in the raw materials for which we can obtain proposed mineralogical composition, lower process temperatures, and the ease of grinding operation, the CSA clinker is considered a potential substitute for OPC cement [16,17]. 

Therefore, the aim of our study is to investigate the influence of slag and fly ash additions to CSA on compressive strength developed after 2, 7, and 28 days. 

## 2. Materials and Methods 

Five different clinker compositions where designed and sintered at 1300 °C from different raw materials. The strength evolution was investigated based on the ye’elimite content. From the clincher with the higher strength were designed several Portland cements based on the EN-197 cement standard. The clinker was previously ground at 3000 cm^2^/g before performing the mechanical testing.

In this study, the following materials were used. For the clinker: limestone, clay, gypsum, and bauxite. For cement development: limestone, slag, and fly ash. The oxide composition of the raw materials and clinker is presented in Table 1. The same clinker was used for all cements, with the default mineralogical composition (Table 1).

The raw mix was dry-homogenized. For the synthesis, we used finely ground limestone filler, with a maximum residue on the 200 μm sieve of 2% and of 20% on the 90 μm sieve. The clay, gypsum, and bauxite were previously crushed and ground in the planetary ball mill (Fritsch Pulverisette planetary ball mill, Idar-Oberstein, Germany). The raw materials were dosed and homogenized in a planetary ball mill for 1 h at 200 rotations per minute in batches of 300 g. The mixture was ground and homogenized in a planetary ball mill to a fineness of R_009_ = 14%. The limestone used was a commercial limestone filler with 5000 cm^2^/g. The obtained raw meal was shaped into green pallets by uniaxial pressing with a pressure up to 150 MPa.

In order to choose the most relevant sintering temperature, a differential scanning calorimetry analysis was performed, and after the analysis, a clinker temperature of 1300 °C was chosen. The raw mix was treated at 1300 °C with a heating rate of 10 °C/min and a sintering time of 30 min, followed by air quenching. 

**Figure 1:**
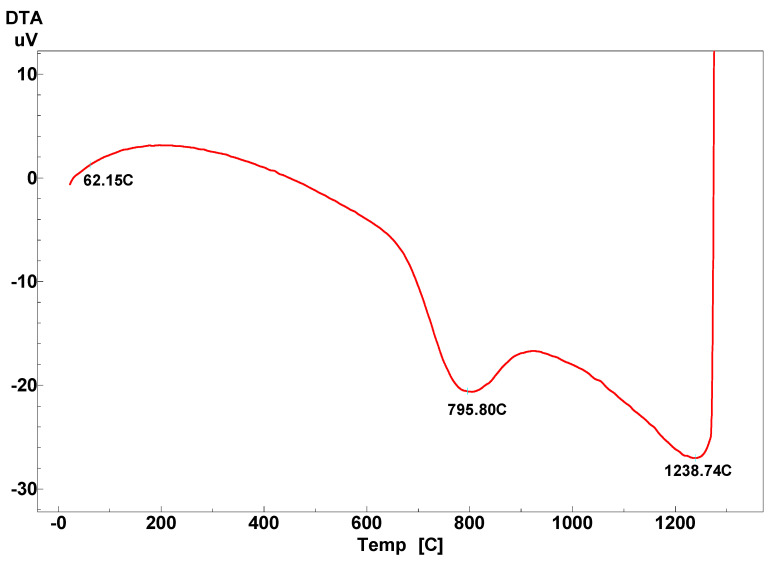
Differential scanning calorimetric analysis for the raw mix.

Figure 1 shows the differential scanning calorimetric curves for the raw mixture, limestone, clay, gypsum, and bauxite. The mixture was heated to a temperature up to 1300 °C at a rate of 10 °C/min. Below 100 °C, a very low endothermic effect originated from the evaporation of the physical water in all the raw materials. An endothermic effect at approx. 800 °C was observed and associated with the decarbonation of calcium carbonate. An exothermic effect that occurred at a much higher temperature (approx. 1240 °C) can be attributed to the reaction of formation and crystallization of the compounds of interest.

The hydration properties were investigated by X-ray diffraction (Shimadzu diffractometer XRD 6000, Shimadzu, Kyoto, Japan) and thermal analysis (Shimadzu DTG-TA 51H, Shimadzu, Kyoto, Japan) on cement mixtures containing clinker, limestone slag, and fly ash. 

Cements were prepared, according to European cement norm, although using CSA clinker [18], through simultaneous grinding of the components. The grinding was done until the targeted fineness was achieved in a planetary ball lab mill (Fritsch Pulverisette planetary ball mill, Idar-Oberstein, Germany). 

Taking into consideration that gypsum is easier to grind compared with other components and is characterized by an advanced fineness, it was separately ground and added in the finishing step to ensure an appropriate cement homogenization and a reduced energy consumption; a final Blaine specific surface area of about 3.000 cm^2^/g was achieved with a planetary laboratory mill (Fritsch Pulverisette planetary ball mill, Idar-Oberstein, Germany). The clinkers were ground in batches of 50 g for 6 min at a speed of 150 rot/min.

The prepared cements are described and named as following:

(a) CI—CEM I, according to [18], from the clinker obtained previously, used an additional 5% natural gypsum ground at a fineness of 3061 cm^2^/g. 

(b) CIIAS—CEM II AS, according to [18], was prepared as a mix of clinker with 10% slag (which was previously ground, up to the specific surface of 1177 cm^2^/g and with 5% gypsum (3061 cm^2^/g)). 

(c) CIIBM—CEM II BM (S-V) type cement was prepared using the clinker with 15% fly ash, 13% GGBF slag, and 5% gypsum. The fly ash fineness was 1082 cm^2^/g.

The hydration processes were investigated on pastes prepared with a 0.5 water/cement ratio and hardened for a period of 2, 7, and 28 days. After curing for 24 h at 20 °C and 95% relative humidity, the samples were demolded and cured in water at 20 °C. The non-standard mold is a cylinder with a diameter of 50 mm and a height of 30 mm.

The mechanical strengths were investigated on mortars according to cement norms [18] for 2, 7, and 28 days’ hardening time. These were determined on three prismatic specimens (40 mm × 40 mm × 160 mm) for each hydration term; thus, for the calculation of average compressive strength, a minimum of 6 compressive strength values were considered, and the deviated values with ±10% were not considered in calculation. The strength tests were performed using a Matest laboratory testing machine (Matest, Treviolo, Italy).

The phase composition of the materials was assessed by X-ray diffraction (XRD) and thermal analysis (DTA-TG).

The X-ray diffraction (XRD) analyses were performed using a Shimadzu XRD 6000 diffractometer (Shimadzu, Kyoto, Japan)with Ni filtered Cu Kα radiation (λ = 0.1054 nm), 2θ ranging between 10 to 80°, with 2°/min, 0.02 min/step. 

A Shimadzu DTG-60 (Shimadzu, Kyoto, Japan) was used to perform the thermal analysis at 30–1000 °C temperature range, 10 °C/min rate of heating, in air. 

The morphological and microstructural characteristics and elemental composition of the samples were determined through scanning electron microscopy (SEM), HITACHI S2600N (HITACHI, Takyo, Japan), coupled with energy dispersive X-ray spectroscopy (EDX) (HITACHI, Takyo, Japan); also, the samples were coated with a thin silver layer.

In order to evaluate the hydration processes, cement pastes were prepared to investigate the mineralogical composition and morphology, by stopping the hydration at different ages.

## 3. Results and Discussion

### 3.1. Clinker Characterization

#### 3.1.1. XRD Analysis

**Figure 2:**
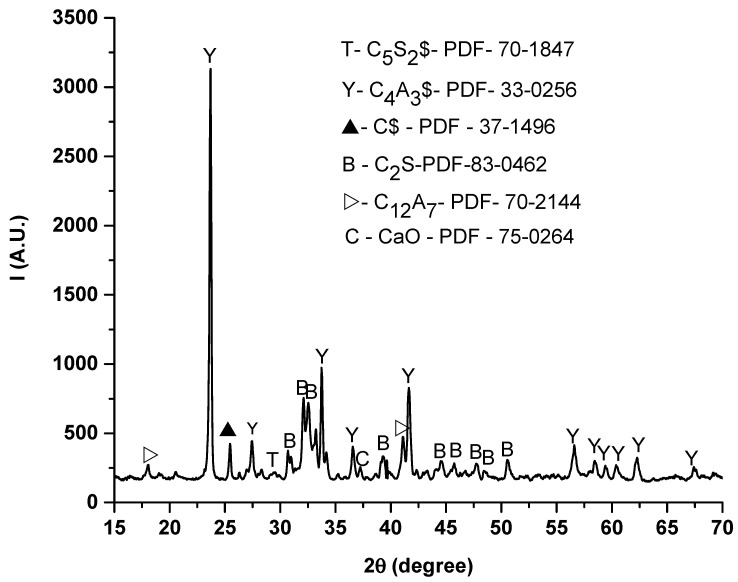
X-ray diffraction pattern on obtained clinker at 1300 °C. (C-CaO, S-SiO_2_, A-Al_2_O_3_, $-SO_3_).

Figure 2 highlights the X-ray diffraction pattern on sintered clinker at 1300 °C. As it can be seen, several crystalline phases are present, three of which as major phases: ye’elimite (C4A3$, PDF- 33-0256), belite (C2S, PDF- 83-0462), and anhydrite (C$- PDF- 37-1496). Therefore, the clinker shows a good hydraulic activity, with potential high early-stage compressive strength due to the ye’elimite, and late-stage strength due to the belite. The anhydrite is present from the dehydration of the gypsum and, during the clinkerization process, plays an important role in increasing the ye’elimite amount.

A slight diffraction peak of ternesite (PDF -70-1847) is present. Early studies [19] are suggesting that C_2_S is reacting with anhydrite to form ternesite in certain conditions. Minor phases typical of raw clinker mixtures are present, such as C_12_A_7_ (PDF- 70-2144) and free lime (PDF- 75-0264).

#### 3.1.2. SEM-EDS Analysis

**Figure 3:**
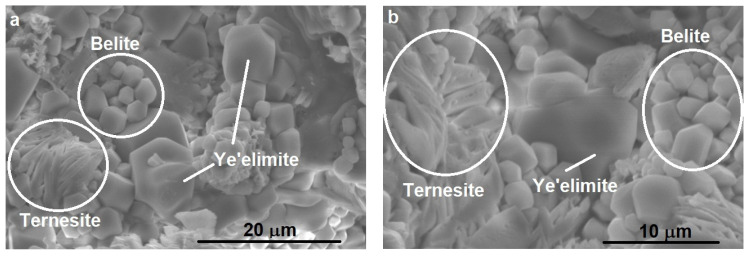

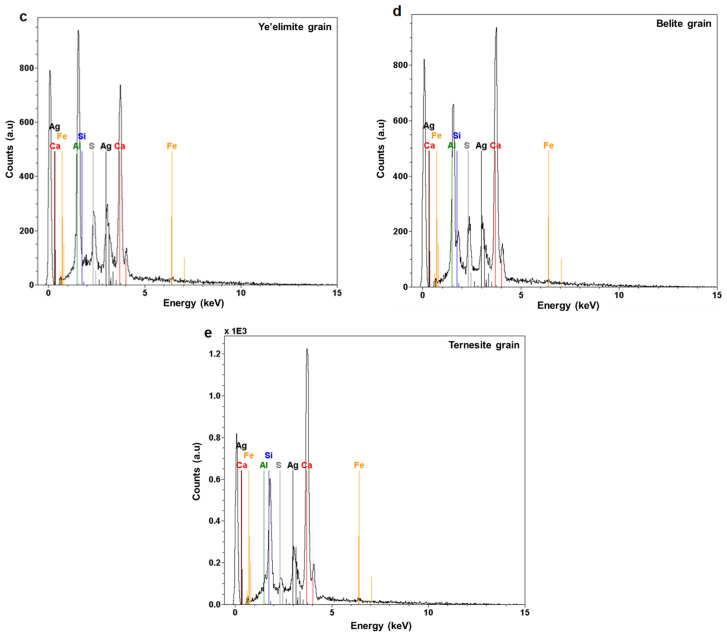
Scanning electron microscopy images (**a**—x2.5k, **b**—x4k) associated with EDX analyses (**c**–**e**) on sintered clinker at 1300 °C.

Scanning electron microscopy was performed on the clinker (Figure 3) in order to highlight the morphology of the mineralogical phases, but also to be able to evaluate the approximate size of them. The EDX spectra were also drawn in order to be able to identify the elemental composition and, implicitly, the mineralogical phases. Figure 3 shows that a mixture of large and small grains is with different morphologies is present in the clinker. According to the SEM analysis, the clinker consists of hexagonal crystals of ye’elimite, as is reported [20], and rounded crystals of belite [12,21].

Knowing that the ye’elimite (C_4_A_3_$) consists of 50% Al_2_O_3_, 13% SO_4_, and 36% CaO, from the EDX spectra of the areas on elements we can identify the larger grains with smooth appearance as very rich areas in Al, which can be attributed to calcium sulfoaluminate. Also, the EDX spectrum on the large grains has a higher proportion of aluminum compared to the small grains (Figure 3a).

The small grains can be identified from the mapping with Si, the silicon being mainly concentrated in the areas with small granules and knowing that C_2_S has 34% SiO_2_ and 65% CaO, so these small grains can be attributed as C_2_S content. This fact is also confirmed by the EDX spectrum drawn for small grains, where the highest proportion of Ca is highlighted.

The undefined “flake” granules can be attributed to the formation of ternesite (C_5_S_2_$) having 25% SiO_2_, 16% SO_4_, and 58% CaO, and according to the EDX spectrum, it has a low S content and a high Ca content. 

### 3.2. Cements Characterization

#### 3.2.1. XRD Analysis

**Figure 4:**
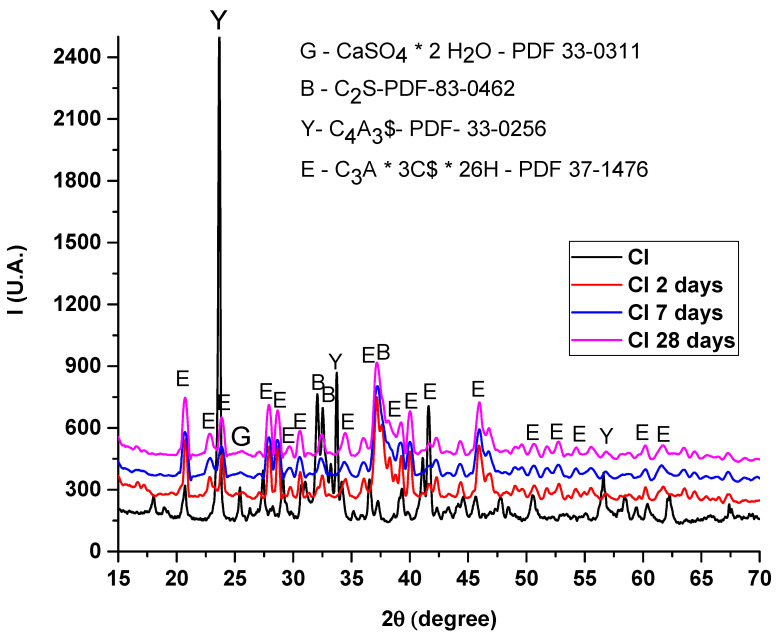
X-ray diffraction pattern on sintered clinker and different curing time. (C-CaO, S-SiO_2_, A-Al_2_O_3_, $-SO_3_, H-H_2_O).

From the point of view of the hydration processes, Figure 4 shows X-ray diffraction patterns at different hydration times of clinker blended with gypsum compared with CI. According to the X-ray diffraction files, the diffraction patterns correspond to a mixture of ettringite (PDF 37-1476) with low quantities of belite (PDF 83-0462) and unreacted ye’elimite (PDF 33-0256) at early-stage hydration. The ye’elimite dissolution and ettringite formation occurs at an early stage due to the hydration kinetics of the added calcium sulfates. By adding gypsum, an early dissolution is favored. The kinetics of ye’elimite hydration are still under investigation [22,23], but these results consider that calcium sulfate has a critical role in controlling the kinetics of the hydration reaction. Previous studies [24] point out that the maximum point of hydration for a ye’elimite based clinker blended with gypsum is registered after 8 h of hydration.

#### 3.2.2. TG-DTG Analysis

Also, in order to investigate the hydration process, thermal analyses were performed.

Thermal analysis for CI and CIIAS pastes hydrated at different ages were performed. The mass loss according to temperature and the endothermic effects are highlighted. Using the thermogravimetric curves, it is possible to emphasize the formation of Al (OH)_3_, which results from the hydration of the ye’elimite. The hydration results predominantly in an amorphous aluminum hydroxide, difficult to detect by the X-ray diffraction. The hydrated cement paste’s thermal behavior is similar. The thermal loss can be appreciated around 25% in the temperature range of 30–1000 °C.

**Figure 5:**
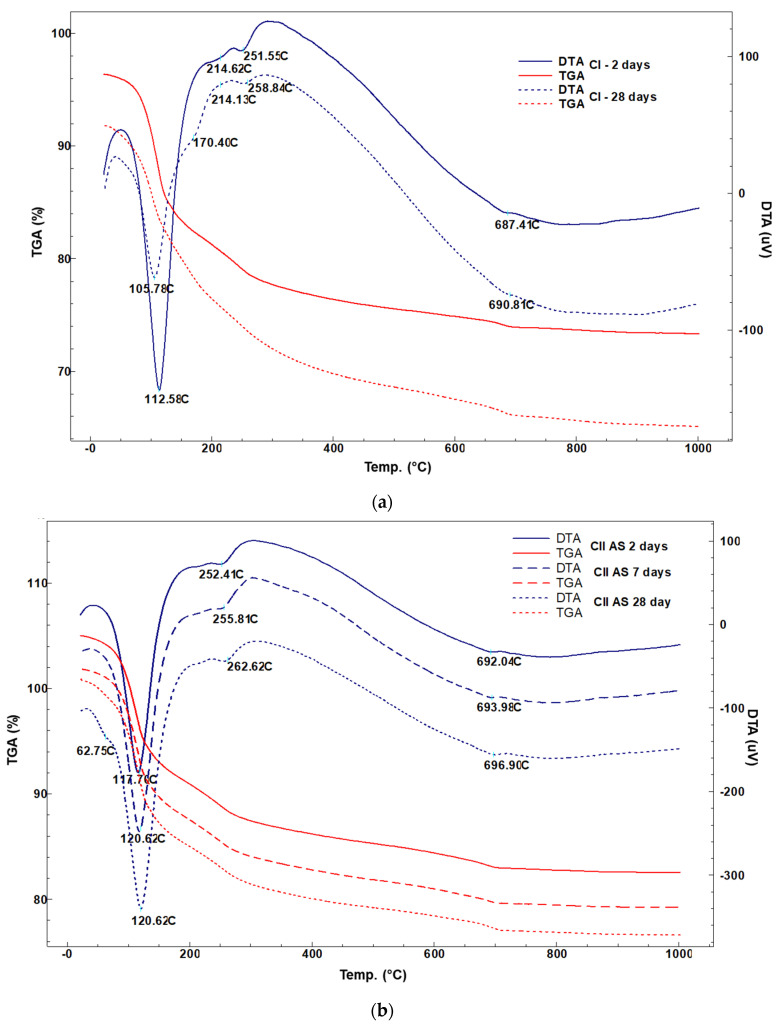
Thermal analysis of hydrated cement pastes for 2, 7 and 28-days: (**a**) CI, (**b**) CIIAS.

The endothermic effects in the temperature range of 30–120 °C typically belong to processes of physically bound water loss and of dehydration of ettringite (117–121 °C) and gypsum (60 °C, 110 °C). In the temperature range 250–280 °C, aluminum hydroxide loses the chemical bonded water. At higher temperatures, between 680 and 692 °C, an endothermic effect occurs which can be associated to the dehydration of Al(OH)_3_ or its complete dehydration with α alumina formation (Figure 5).

#### 3.2.3. Mechanical Strength

Considering the mechanical tests, the results are presented in Table 2. 

The CI sample had the highest mechanical strength (60.9 MPa at 28 days), which fits to an equivalent of CEM I 52.5 N Portland cement class. As was expected, the compression strength increased with time from 2, 7, and 28 days for all investigated samples. Also, the mechanical properties decrease with the clinker content due to the clinker dilution effect. It is worth mentioning that the fineness of the ground mixture is between 2400–2500 cm^2^/g, corresponding to a very coarse cement. For ordinary Portland cement (OPC), a superior grade of 52.5 is always obtained at a grinding fineness of 4400–4600 cm^2^/g.

#### 3.2.4. SEM-EDS Analysis

In order to follow the influence of the specific grinding surface on the mechanical strengths, the CI cement was ground to a higher specific surface area compared to those required to obtain ordinary Portland cement. A specific surface area of 4600 cm^2^/g was obtained, this having a positive influence on the mechanical strength. The compressive strength at 2 days were most dependent on the fineness of grinding and, as expected, increased by 7 MPa. The specific surface influences, also, the compressive strength at final stages, obtaining 65 MPa at 28 days. For CIIAS, 42 MPa was achieved at a grinding fineness of 3055 cm^2^/g, according to the European standard EN 197-1 A CEM II AS 42.5R. The CIIBM corresponds to CEM II BM (S-V) based on the European norm.

Scanning electron microscopy was used to assess the morpho-structural evolution at different curing time periods of the CI, CIIAS, CIIBM mortars. As reported in previous research, the proportion of ye’elimite is a major factor in the evolution of mechanical strength. The increase of the ratio of ye’elimite will determine a high mechanical strength due to the ettringite formation [22].

**Figure 6:**
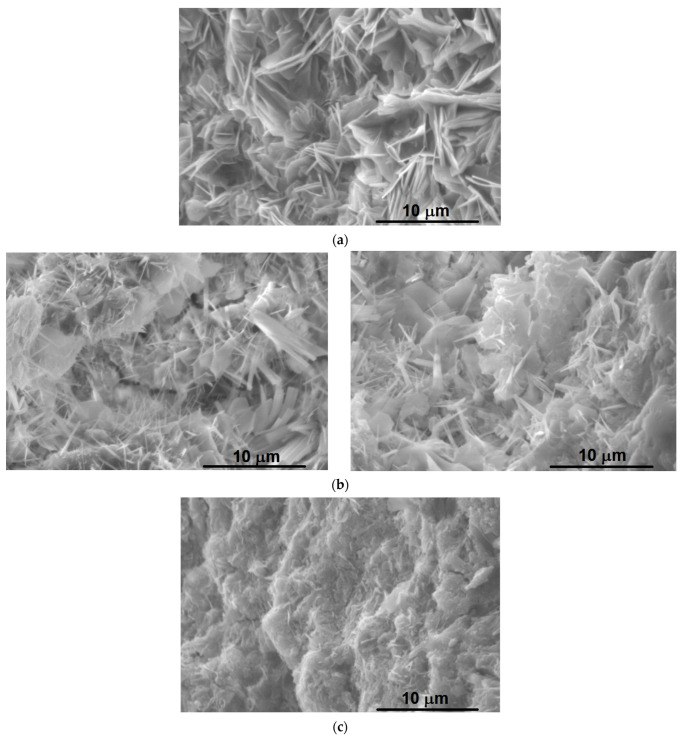

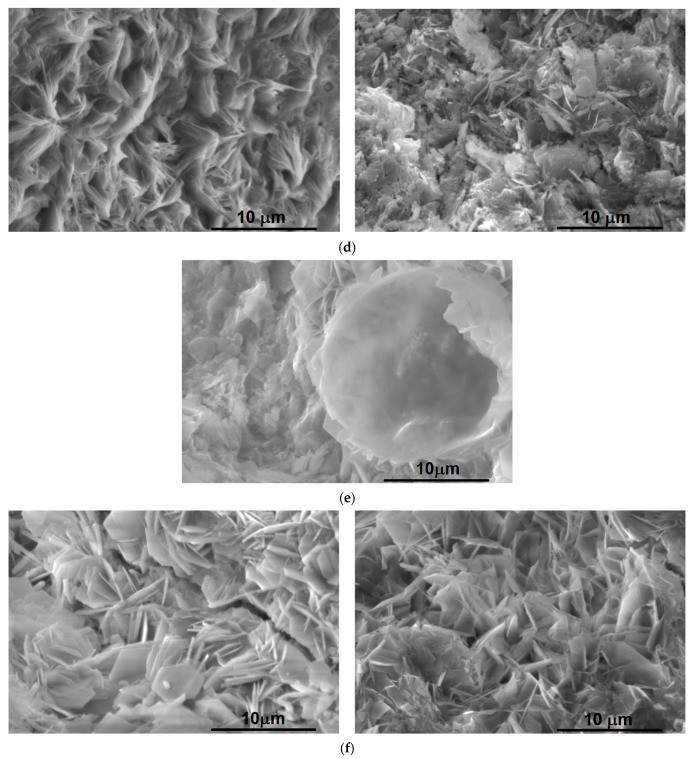
Scanning electron microscopy images performed on mortars at 7 and 28 curing days: (**a**) CI at 7 days; (**b**) CI at 28 days; (**c**) CIIAS at 7 days; (**d**) CIIAS at 28 days; (**e**) CIIBM at 7 days; and (**f**) CIIBM at 28 days.

Figure 6 shows scanning electron microscopy images for cured mortars at different stages; the microscopy was performed on the mortar piece obtained for compressive strength testing.

The mortar obtained from the clinker, mixed with gypsum, CI hydrated 7 days displays the formation of crystal plate-like particles specific to ettringite due to high content of sulfate [25]; at 28 days, a specific morphology of calcium sulphosilicate hydrates and calcium silicate hydrates mixed with ettringite and, possibly, stratilingite is highlighted [4].

CIIAS cement mortar has a morphology of calcium silicate hydrates in the form of fibers at 28 days. At 7 days, a mixture of portlandite (hexagonal plate crystals) and ettringite is highlighted.

CIIBM cement mortar obtained is a mixture of 15% fly ash and 13% slag. The SEM images show, after 7 days, the unreacted fly ash grains (broken sphere) and a high quantity of portlandite, which could explain the lower compressive strength value, as compared to CIIAS and CI (Figure 6).

Etrringite is a phase whose presence is easily demonstrated by SEM images. This phase is responsible for the early hydration of sulfoaluminate cement and has major role for the development of high early strength. It is preferred as hydration form, and it can appear even in low sulphur environment.

The SEM images acquired on the samples aged for 7 days show a distinctive surface morphology of crystallite-like structures densified probably due to monosulfate and hydrosilicate formation (Figure 6a). A tendency toward surface granulation along the crystallites can be noticed when slag was introduced into the composition (Figure 6c). The supplementary addition of fly ash to the CIIBM mortar could lead to the broken shell-type structure (Figure 6e). In early stages, it can be noticed that fly ash cenospheres and some Portlandite crystals generated by the belite hydration (Figure 6e,f). Due to the hydraulic activity of the fly ash, it can be assumed that part of the Portlandite is consumed.

The increase of the curing time generates the occurrence of needle-like shapes through the crystallites present on CI sample due to AFm in to ettringite transition. (Figure 6b). Moreover, an increasing aging time of the mortars induces a crystallization effect regardless of the composition involved (Figure 6d,f).

## 4. Conclusions

This paper focus is on the synthesis of CSA starting from CSA clinker obtained from the natural raw materials such as limestone, clay, bauxite, and gypsum. The obtained mortars were investigated to understand the influence of clinker and additives nature on CSA mortar properties. The clinker was obtained at 1300 °C and was investigated by XRD and SEM to determinate the phase composition and morphology. The mortars obtained from the grinded clinker with gypsum and slag were evaluated in terms of mechanical properties, which revealed the important influence of the clinker factor, mineralogical composition, and specific surface area. The clinker replacement is up to 10% concentration with slag from industrial waste, obtaining good mechanical properties. 

The hydration of sulphoaluminate cements on cement pastes have been studied, revealing that the main hydration products for hydrated CSA clinkers are ettringite, monosulfate, and Al(OH)_3_, according to the reaction (2). The ettringite formation also strongly depends on the dissolution of gypsum. Ettringite is a well crystallized mineral phase whose presence is easily demonstrated by X-ray diffraction analysis and SEM investigations. This phase is important in the early hydration of sulfoaluminate cement and is relevant for the development of high early-stage compressive strength. The importance of ettringite is clearly demonstrated in terms of early age behavior.

The obtained results, according to the SEM analysis, show that the clinker consists of hexagonal crystals of ye’elimite and rounded crystals of belite.

From the point of view of hydration processes, according to the X-ray diffraction files, the diffraction patterns correspond to a mixture of ettringite, with low quantities of belite, and also unreacted ye’elimite, at early-stage hydration. The ye’elimite dissolution and ettringite formation occurs at early stage due to the hydration kinetics of the added calcium sulfates. By adding gypsum, an early dissolution is favored. Although the kinetics of ye’elimite hydration are still under investigation, the obtained results emphasize that calcium sulfate has a critical role in controlling the kinetics of the hydration reaction.

Mortars prepared using the CSA clinker show higher compressive strengths, due to the high ye’elimite and belite contents. The CSA cement showed a significant reaction with high mechanical strengths, 60.9 MPa at 28 days which fits to an equivalent of CEM I 52.5 N Portland cement class.

## Figures and Tables

**Table 1:** Chemical compositions of raw materials used, and the designed mineralogical composition of CSA.

Chemical	Raw Materials								Clinker		
	Limestone [wt %]		Gypsum [wt %]		Clay [wt %]	Slag [wt %]	Fly ash [wt %]	Bauxite [wt %]	[wt %]		
	CaCO_3_	99.5	CaSO_4_۔2H_2_O	83.53					Chemical	Mineralogical	
CaO	97.2		34.5		3.33	3.8	41.8	4.5	0.3	C4A3$	45
Na_2_O	0.0		0.0		0.9	1.0	0.6	0.5	0.1	C5S2$	20
MgO	0.2		0.4		2	2.3	7.1	2.0	0.4	C2S	20
Al_2_O_3_	0.9		0.5		18	20.6	7.7	25.5	89.0	C4AF	8
SiO_2_	1.6		2.1		53	60.5	41.4	55.7	5.7	C$	7
SO_3_	0.0		62.1		0.2	0.2	0.6	0.9	1.2		
K_2_O	0.1		0.1		3.13	3.6	0.6	2.6	0.8		
Fe_2_O_3_	0.0		0.3		7	8.0	0.2	8.2	2.5		

**Table 2:** Compressive strength of mortars at different hydration times.

Sample		CI	CIIAS	CIIBM	CP
SSP Blaine (cm^2^/g)		2580	3055	2676	4600
Compressive strength (MPa)	2 days	31.6	33	22.9	37.2
	7 days	31.5	39.5	27.2	-
	28 days	60.9	42	43	65

## Data Availability

Not applicable.
